# Biophysical Characterization of a Novel Tri-Layer Placental Allograft Membrane

**DOI:** 10.1089/wound.2020.1315

**Published:** 2021-11-11

**Authors:** Paul P. Bonvallet, Sita M. Damaraju, Heli N. Modi, Victoria L. Stefanelli, Qiaoling Lin, Sunil Saini, Ankur Gandhi

**Affiliations:** Product Development, Integra Life Sciences, Corp., Princeton, New Jersey, USA.

**Keywords:** placental membranes, chronic wounds, wound healing

## Abstract

**Objective:**

Placental tissues, including membranes composed of amnion and chorion, are promising options for the treatment of chronic wounds. Amnion and chorion contain multiple extracellular matrix (ECM) proteins and a multitude of growth factors and cytokines that, when used clinically, assist in the progression of difficult to heal wounds through restoration of a normal healing process. The objective of this study was to characterize the *in vitro* physical and biological properties of a dehydrated tri-layer placental allograft membrane (TPAM) consisting of a chorion layer sandwiched between two layers of amnion.

**Approach:**

Mechanical properties were evaluated by mechanical strength and enzyme degradation assays. The ECM composition of TPAM membranes was evaluated by histological staining while growth factors and cytokine presence was evaluated by a multiplex enzyme-linked immunosorbent assay. Proliferation, migration, and ECM secretion assays were performed with fibroblasts. Immunomodulatory properties were assessed by a pro-inflammatory cytokine reduction assay while the macrophage phenotype was determined by quantifying the ratio of M1 versus M2 secreted factors.

**Results:**

The unique three-layer construction improves mechanical handling properties over single- and bi-layer membranes. Results demonstrate that TPAM is rich in ECM proteins, growth factors, cytokines, and tissue inhibitors of metalloproteinases, and favorably influences fibroblast migration, proliferation, and ECM secretion when compared to negative controls. Furthermore, after processing and preservation, these membranes maintain their intrinsic immunomodulatory properties with the ability to suppress pro-inflammatory processes and modulate the M1 and M2 macrophage phenotype toward a pro-regenerative profile when compared to a negative control.

**Innovation:**

This is the first study to characterize both the biophysical and biological properties of a tri-layer placental membrane.

**Conclusion:**

This work demonstrates that TPAM has improved handling characteristics over single- and bi-layer membranes, stimulates pro-healing cellular responses, and advantageously modulates inflammatory responses, altogether making this scaffold a promising option for treating wounds, especially those that are complex or difficult to heal.

**Figure:**
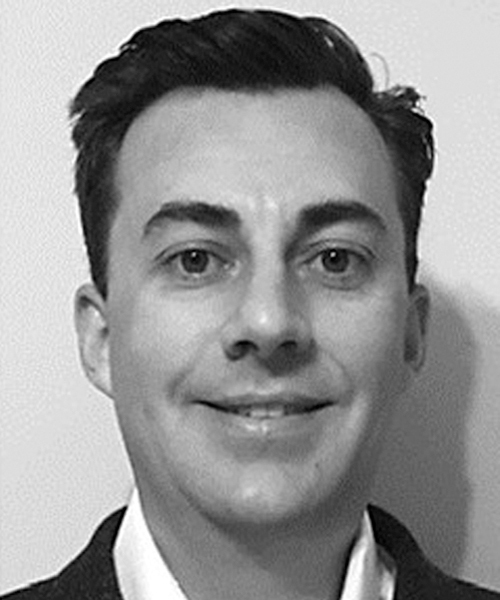
Paul Bonvallet, PhD

## Introduction

Diabetic foot ulcers, pressure ulcers, and venous leg ulcers are chronic wounds that are defined by their persistent, nonhealing state. In the United States, an estimated $20 billion is spent annually for the management of these chronic wounds.^1^ Chronic wounds disproportionately impact the elderly presenting with underlying conditions of vascular disease, diabetes, and other comorbidities. Treating these wounds is challenging due to underlying pathologies such as impaired blood flow, increased exudate production, infection, prolonged presence of immune cell responders, continual matrix breakdown, and an excessive expression of pro-inflammatory cytokines, reactive oxygen species, and proteases.^2–4^ Treatments such as wound debridement, topical ointments, dressings, negative pressure wound therapy, dermal substitutes, and recombinant growth factors have been used, but these are limited in success.^5,6^ Therefore, a treatment option that can promote a multifaceted signaling response to address the complex pathophysiology and prolonged inflammation is likely necessary for progressing chronic wounds to resolution.^7^

Placental tissues have shown to be an efficacious option for treating chronic wounds.^7^ More specifically, in the last 30 years significant progress has been made to deconstruct and preserve placental tissues for clinical use. The membranes most commonly used clinically are derived from the placenta consisting of two distinct layers, an amnion and chorion that are known to be immune privileged. The amnion is the innermost thin membrane that is ∼35–60 μm thick and is composed of five layers including an epithelium, thick basement membrane, compact layer, fibroblast layer, and spongy layer. The chorion is a considerably thicker membrane that is ∼200–300 μm thick and consists of fibroblast and basement membrane layers.^8^ These membranes contain a unique combination of extracellular matrix (ECM) proteins and signaling molecules involved in tissue growth and regeneration. Specifically, they contain proteins such as collagen types I and III, fibronectin, and laminin and possess over 280 soluble biomolecules.^9–12^ Some of the marked features of these growth factors are their ability to influence proliferation, migration, and ECM secretion from cells such as fibroblasts, in addition to support angiogenic, anti-inflammatory, and immunomodulatory functions.^13–16^ Clinical case studies utilizing placental membranes for up to 8 weeks in burns and complex wounds as a biological dressing have demonstrated decreased inflammation, reduced pain and scarring, and enhanced re-epithelialization.^17,18^ The inherent bioactive properties and favorable clinical findings further reinforce the therapeutic potential of placental membranes as a therapy for chronic wound healing and tissue repair.

**Figure 1.**
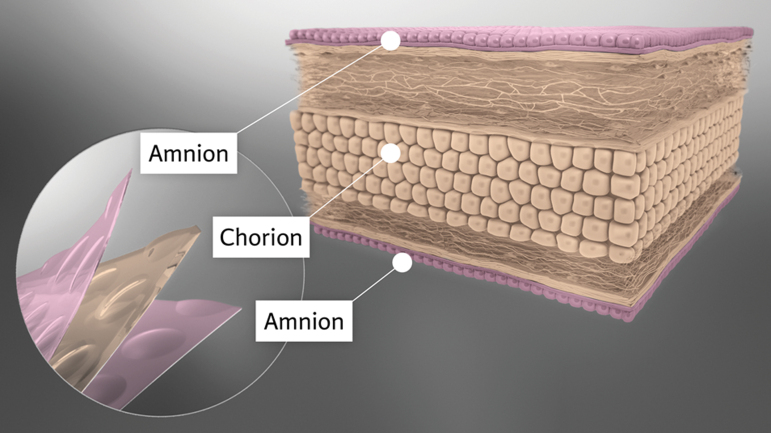
TPAM is a three-layer membrane (amnion, chorion, amnion). Multiple layers provide a robust strength and degradation profile. Processing methods preserve the proteins and signaling factors present within TPAM. Factors preserved within TPAM favorably influence cell migration, cell proliferation, ECM secretion, and immune cell modulation. ECM, extracellular matrix; TPAM, tri-layer placental allograft membrane. Color images are available online.

Placental allografts undergo preservation techniques that include dehydration, cryopreservation, or hypothermic storage and are designed to include a range of membrane configurations from amnion as a single layer, to amnion and chorion as a bi-layer wound dressing.^19–21^ Preservation techniques and manufacturing processes have a significant impact on biomolecule stability, biophysical properties, and bioactivity of the tissues that ultimately affects clinical outcomes.^22–24^ Dehydrated amniotic membranes have previously been shown to have a higher bioavailability and growth factor content compared to cryopreserved allografts.^24^ Other studies have investigated the *in vitro* biological efficacy of amnion alone and amnion/chorion membranes using different cell lines to demonstrate wound healing applications.^9,16,25,26^ However, the biophysical properties of these various membranes can limit their clinical utility. When hydrated, a placental membrane often becomes self-adherent and lacks mechanical rigidity that results in compromised handling properties and is difficult to position on a wound bed. Single and bi-layer membranes have limited residence time, affecting the ability to deliver sustained bioactive molecules into the wound environment. Clinically, an increased residence time would result in a reduced number of applications,^22^ resulting in a reduced overall health care cost. An improved residence time also has benefits for internal surgical applications where only one application is possible. These shortcomings can be addressed with a tri-layered membrane, tri-layer placental allograft membrane (TPAM) (Fig. 1). TPAM, consisting of three layers, is a non side-specific membrane with enhanced handling properties that enables a range of clinical applications including chronic, deep, and irregularly shaped wounds. Furthermore, the thicker membrane can be easily positioned or repositioned within a wound for improved conformity.

The objective of this study was to characterize the biophysical properties of TPAM consisting of amnion-chorion-amnion layers as compared to single- and bi-layer membranes. Further analyses include biochemical composition and effects of TPAM on *in vitro* cellular responses as it relates to wound healing. Specifically, biophysical properties were evaluated by tensile testing and *in vitro* degradation via an enzyme degradation assay. TPAM compositional analysis included histology for ECM and a multiplex enzyme-linked immunosorbent assay (ELISA) for growth factors and cytokines. *In vitro* bioactivity of human fibroblasts for wound healing was evaluated in the presence of TPAM extracts. Finally, the immunomodulatory properties were evaluated by inflammation inhibition and macrophage polarization assays.

## Clinical Problem Addressed

Chronic wounds present a significant burden to the medical system both in terms of finance and patient long-term health outcomes and quality of life. Amnion and chorion wound dressings provide a bioactive solution that aides in the progression of difficult to heal wounds through the inflammatory phase of wound healing. Clinically, it would be advantageous to have a product that can withstand a harsh chronic wound environment while also delivering a sustained growth factor supply to favorably alter the local immune environment. The data presented herein demonstrate that TPAM provides robust mechanical and bioactive properties that have potential to promote favorable resolution of chronic wounds.

## Materials and Methods

All data were recorded in an electronic lab notebook (elabjournal.com).

### TPAM membranes and extraction

Placental membranes were donated from consenting mothers and collected after live births in accordance with the United States Food and Drug Administration (FDA) and the American Association of Tissue Banks guidelines. Placental tissue recovery and processing is performed by BioDlogics, LLC. BioDlogics, LLC is registered with United States FDA as a manufacturer and Integra LifeSciences Sales, LLC as a distributor of human cells, tissue, and cellular and tissue-based products. Donors were verified negative for communicable disease agents before processing. TPAM (AmnioExcel^®^ Plus; Integra LifeSciences, Princeton, NJ) is a dehydrated tri-layer membrane consisting of a chorion layer imbedded between two layers of amnion. The composite membrane is constructed from a single donor of the placental tissue, processed aseptically with a mechanical cleaning process, and without the use of fixatives. TPAM is stored at room temperature with a shelf-life of up to 5 years. Homogenization of TPAM was executed with 5°cm^2^ tissue per 1 mL of fibroblast basal media (ATCC) with a homogenizer (Precellys; Bertin Technologies). The homogenized material was then extracted at 37°C for 72 h and the supernatant was filtered using 0.22 μm sterile filters (Millipore; Sigma Aldrich).

### Histology

Histological procedures have been described previously in detail.^16^ Briefly, three different lots of TPAM were rehydrated in phosphate buffered saline (PBS) before histology processing. TPAM was then embedded in paraffin and sectioned. Sections were processed with hematoxylin and eosin (H&E, S3301; Dako) and Alcian Blue (Anatech) stains. Sections were processed for immunostaining of fibronectin (Rabbit polyclonal, ab6584; Abcam), collagen type I (Rabbit polyclonal, 2150-0020; Bio-Rad), collagen type III (Rabbit polyclonal, 2150-0100; Bio-Rad), or laminin (Rabbit polyclonal, Z0097; Agilent). Representative images were taken at 40 × with a Confocal Microscope (Leica).

### Growth factor and cytokine compositional analysis

Growth factors and cytokines were quantified in TPAM extracts using a Luminex ELISA assay (Millipore). TPAM was extracted as described earlier, and growth factors and chemokines were quantified in the supernatant and analyzed using a Luminex xMAP technology.

### Mechanical properties

#### Tensile strength

Tensile testing was performed using an Instron 5544 system for single-layer (amnion only, Amnioexcel; Integra LifeSciences), bi-layer (amnion and chorion; Mimedx, Marietta, GA), and TPAM membranes. Samples were cut into 1 × 1 inch length standard dog bone-shaped strips with uniform edges and were then hydrated in distilled water for 20 min before testing (means shown represent independent experiments in triplicate, results shown are from two independent experiments). Samples were clamped at the top and bottom and pulled to break at a rate of 5 mm per min with a load range of 10 N. Maximum loads were recorded at the breakpoint using Blue Hill Instron software.

#### Enzyme degradation

*In vitro* TPAM degradation was evaluated using a cocktail of collagenase (Sigma Aldrich) and thermolysin (Sigma Aldrich) enzymes. Briefly, single-layer, bi-layer, and TPAM membranes were cut into circular 12 mm diameter discs with a biopsy punch (Miltek) and placed into a 20-mL glass vial (means shown represent independent experiments with *N* = 4, results shown are from two independent experiments). 15 mLmilliliters of enzyme solution containing 54 U/mL collagenase enzyme and 3.7 U/mL thermolyzing enzyme were added at pH 7.5 to the vial and warmed to 37°C. Images were taken every 30 min until membranes had completely dissolved. To validate complete membrane dissolution, samples were filtered and reacted with ninhydrin-hydrindantin solution at 100°C for 25 min, diluted with 5 mL 50% v/v solution of n-propyl alcohol, and absorbance were measured on a ultraviolet-Visible spectrophotometer at 570 nm (SpectraMax M3; Molecular Device).

#### Protein release

Total protein release over 28 days was performed in sterile low binding siliconized microcentrifuge tubes (Fisher Scientific). Briefly, 6 cm^2^ of single-layer (three lots, *n* = 3 each lot, total *n* = 9) and 4 cm^2^ of TPAM was placed in tube with 0.5 mL sterile PBS and incubated at 37°C on an orbital shaker at 150 rpm. At each time point, tubes were spun down at 10,000 rpm for 5 min at 4°C. Spent PBS was collected and replaced with 0.5 mL of fresh sterile PBS. The samples were incubated for the next timepoint. The collected supernatant was stored in −80°C until the study completion. A standard BCA assay (Thermo Scientific) was performed to quantify the release of proteins from single-layer and TPAM over a 28-day time period. The total protein (μg) was then normalized to membrane surface area (cm^2^).

### Cellular migration

A standard wound healing scratch assay was executed with human dermal fibroblasts (hDF; ATCC). Briefly, passage 4 hDF were seeded at 70,000 cells per well into a culture-insert (2 well, 24-well plate Ibidi) for overnight cell attachment. After 24 h, inserts were removed, cells were rinsed with PBS, and stained with Hoechst 33342. Treatment groups were added to each well (means shown represent independent experiments in triplicate, results shown are from two independent experiments). Treatments included complete media with 10% fetal bovine serum (FBS) (+ control), basal media with 1% FBS (− control), and TPAM extract conditioned media with 1% FBS at a concentration of 0.75 cm^2^/mL. At 0, 6, 20, and 27 h, images were acquired and analyzed using ImageJ software (National Institutes of Health).

### Cellular proliferation

#### CyQuant cell proliferation assay

Passage 4 hDF were seeded at 800 cells per well in a 96-well plate with treatment groups containing either basal media supplemented with 1% FBS (− control), 0.75 cm^2^/mL TPAM in basal media with 1% FBS, or complete media supplemented with 10% FBS (+ control) and cultured for 7 days (means shown represent independent experiments with *N* = 4, results shown are from two independent experiments). Media was changed once every 2 days and time points were days 1, 3, and 7. CyQuant reagent (ThermoFisher) was added to the wells and fluorescence was assayed using a plate reader (SpectraMax M3; Molecular Device) at 480 nm excitation and 520 nm emission.

#### Cells seeded on TPAM

Passage 4 hDF (240,000 cells/mL) were suspended with TPAM samples (12 mm diameter) in fibroblast growth media in a conical tube. The conical tube was gently agitated on an orbital shaker for 3 h in an incubator at 37°C and 5% CO_2_ for cell attachment. After 3 h, cell seeded TPAM was transferred to a 24-well plate and incubated in fibroblast basal media containing 1% FBS and cultured for 7 days. Fibroblasts were also seeded on tissue culture plastic as a control to validate cell viability. Media was exchanged once every 2 days. At each time point, the samples were fixed using 4% paraformaldehyde in PBS and stained with Alexa Fluor 488 Phalloidin for actin within cytoskeleton and Hoechst 33342 stain for cell nuclei (Thermo Fisher Scientific).

### ECM protein secretion

Passage 4 hDF were seeded at 2,500 cells/cm^2^ in in 24-well plates with treatment groups containing either complete media supplemented with 10% FBS (+ control), basal media supplemented with 1% FBS (− control), or 0.75 cm^2^/mL TPAM in basal media with 1% FBS. Media was changed once every 2 days. Samples were harvested at 7 and 14 days and cells were fixed using 4% formaldehyde, rinsed in PBS, and treated with 0.1% Triton-X100 for 30 min. Cell nuclei (Hoescht 33342) and secreted collagen type I (Bio-Rad) were stained and the imaging was performed using a confocal microscope (Leica).

### Immune modulation

#### Inflammation inhibition

Human peripheral blood mononuclear cells (PBMCs; ATCC) were seeded at 1 × 10^5^ cells per well in a 96-well plate with RPMI 1640 media containing 2 mM l-glutamine, 1% Pen-strep, and 10% FBS (means shown represent independent experiments in triplicate, results shown are from two independent experiments). The cells incubated for 1 h at 37°C. TPAM extracts (concentration = 2.5 cm^2^/mL), lipopolysaccharide (LPS) (+ control), and 1 μM dexamethasone (− control) were added to the wells and incubated for 1 h before addition of LPS for cell stimulation. The plate was incubated overnight, and the supernatants were collected for quantifying pro-inflammatory cytokine release using a Luminex ELISA assay (Millipore HCYTOMAG-60K).

#### Macrophage polarization

Macrophage differentiation and polarization was executed in a manner consistent with the standardizations defined by Murray *et al.*^27^ Briefly, monocytes were isolated from human PBMC (ATCC) via adhesion to tissue culture plastic in a flat-bottomed 96-well plate (5 × 10^5^ cells per well) over a 5-h period in RPMI-1640 media plus 5% FBS (Fisher Scientific). Monocytes were subsequently differentiated into M0 macrophages over a period of 6 days in RPMI-1640 media supplemented at 50 ng/mL with macrophage colony stimulating factor (R&D Systems) and 10% FBS. Polarization stimuli were then incubated with cells for 48 h and included the following: for M1 controls 50 ng/mL interferon (IFN)γ (R&D Systems), 100 ng/mL LPS (Sigma Aldrich), 10% FBS; for M2 controls 20 ng/mL IL-4 (R&D Systems), 10% FBS; for M0 controls basal media consisting of RPMI and 5% FBS; for TPAM 6 mm discs of the construct were allowed to float in basal media. Following polarization, wells were thoroughly washed of all polarizing stimuli, and they were provided 130 μL of fresh basal media per well for collection of secreted factors over the subsequent 24 h (means shown represent independent experiments in triplicate, results shown are from two independent experiments). Cytokine content of supernatants was measured using a Luminex kit (R&D Systems) for analytes including platelet-derived growth factor (PDGF)-B, CCL-22, CCL18, IL-13, tumor necrosis factor (TNF)α, IFNγ, interleukin (IL)-6, and RANTES. This experiment was run twice with *N* = 3 per condition in each run.

### Statistical analysis

GraphPad Prism 8 software was used for data analysis of all quantitative results. Results are expressed as mean ± standard deviation. Statistical significance was determined by performing a Student's *t*-test or a one-way analysis of variance with the *post hoc* multiple comparison using Tukey's test. Probability (*p*) values <0.05 were considered statistically significant.

## Results

### TPAM has three distinct layers

**Figure 2.**
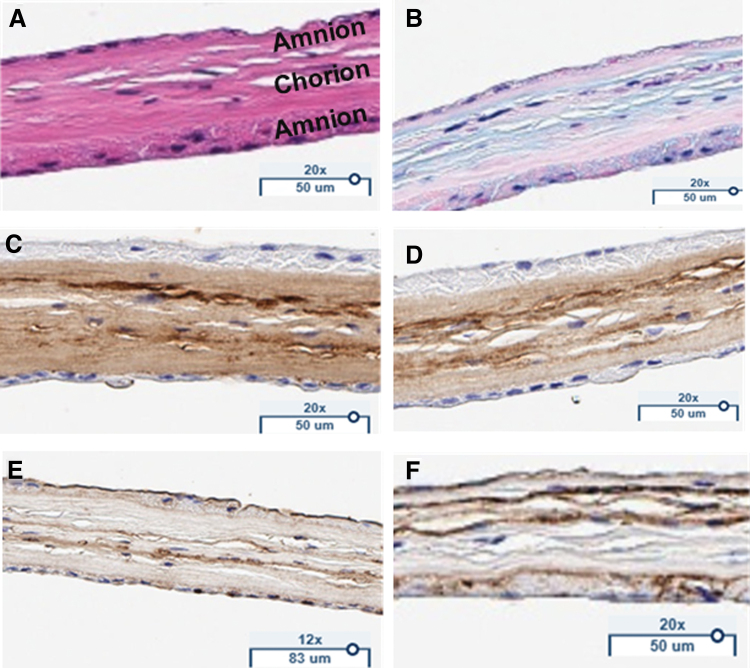
TPAM stained for **(A)** hematoxylin and eosin, **(B)** Alcian Blue, **(C)** Collagen I, **(D)** Collagen III, **(E)** Fibronectin, **(F)** Laminin. Color images are available online.

Histological staining was performed to identify the three distinct layers and the ECM proteins preserved in TPAM. H&E staining (Fig. 2A) reveals three placental layers with two layers of amnion denoted by the epithelial membranes on the upper and lower side of the membrane and a thicker chorion in between. Alcian blue stain (Fig. 2B) reveals the presence of glycosaminoglycans and proteoglycans embedded within TPAM. Furthermore, immunohistochemistry staining (Fig. 2C–F) demonstrates a significant presence of laminin, fibronectin, and collagen types I and III ECM proteins. Histological staining of single- and bi-layer membranes has been previously reported in literature.^17,26^

### TPAM contains endogenous factors relevant for wound healing

ELISAs performed on TPAM extracts (not shown) demonstrate the presence of growth factors and cytokines. Some of these factors include fibroblast growth factor (FGF)-2, PDGF-AA, PDGF-BB, transforming growth factor (TGF)-β1, TGF-β2, TGF-β3, IL-8, epithelial growth factor (EGF), vascular endothelial growth factor (VEGF), tissue inhibitor of metalloproteinase (TIMP)-1, and TIMP-2. These factors are all involved in the complex signaling that occurs throughout the phases of wound healing and all help to progress wounds to resolution.

### TPAM has stronger mechanical properties than single- and bi-layer membranes

**Figure 3.**
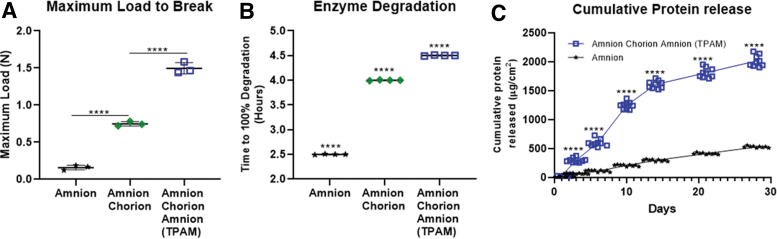
Evaluation of TPAM mechanical properties. **(A)** Maximum load to break of one, two, and three-layer placental membranes (one-way ANOVA, ****p < 0.0001). **(B)** Enzyme degradation assay measuring the time to 100% degradation of one, two, and three-layer membranes (One-way ANOVA, ****p < 0.0001 for all data points compared to each other). **(C)** Protein release assay measuring the total cumulative protein release over a 28-day time period (Two-way ANOVA, ****p < 0.0001 comparing the membranes at each time point). ANOVA, analysis of variance. Color images are available online.

A major shortcoming of amniotic membrane-based products is their lack of mechanical strength leading to compromised handling properties when used in a clinical setting. Figure 3A demonstrates that TPAM has a significantly higher maximum allowable force to break compared to a bi-layer amniotic membrane (twofold; *p* < 0.0001) and single-layer amniotic membrane (fivefold; *p* < 0.0001). An *in vitro* enzymatic degradation assay was performed to compare the structural integrity of TPAM, single-layer, and bi-layered amniotic membranes. Enzymes in this experiment were chosen to mimic an accelerated biological degradation environment that occurs naturally *in vivo.* As shown in Fig. 3B, TPAM had a significantly (*p* < 0.0001) slower degradation time of 4.5 h compared to a bi-layer (4 h) and single-layer membrane (2.5 h) for complete degradation.

### TPAM releases a sustained, higher level of proteins than single-layer membranes

The release of proteins from placental membranes can vary based on membrane configuration. When compared to amnion alone, chorion increases membrane thickness 5–6 times. When chorion is included in placental membranes the release kinetics of protein increases in concentration and time due to membrane thickness. As seen in Fig. 3C, TPAM has a fivefold higher concentration release of proteins than single layer amnion for as long as 14 days. After 14 days, TPAM has sustained lower levels of protein release.

### TPAM promotes a favorable cellular response

**Figure 4.**
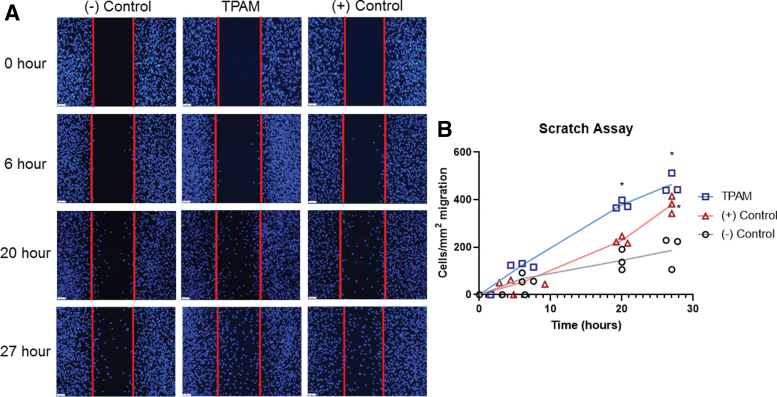
Evaluation of TPAM effect on fibroblast cell migration—Scratch assay. **(A**, **B)** Analysis of hDF migration across a void space of 500 μm at 0, 6, 20, and 27 h (Two-way ANOVA, *p < 0.05 as compared to [−] control). hDF, human dermal fibroblasts. Color images are available online.

**Figure 5.**
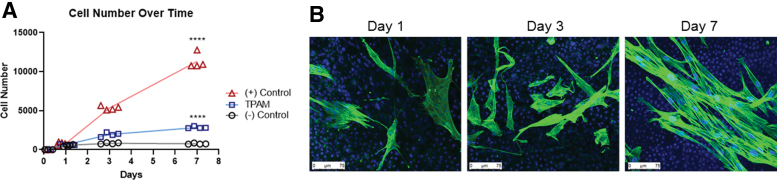
**(A)** A DNA CyQUANT assay shows hDF proliferation over time when treated with TPAM (Two-way ANOVA, ****p < 0.0001 as compared to [−] control). **(B)** hDF seeded on the surface of TPAM membranes and cultured in basal media for 1, 3, and 7 days and stained for actin cytoskeleton (*green*) and nuclei (*blue*). Note that the nuclei from TPAM are also stained *blue*. Color images are available online.

**Figure 6.**
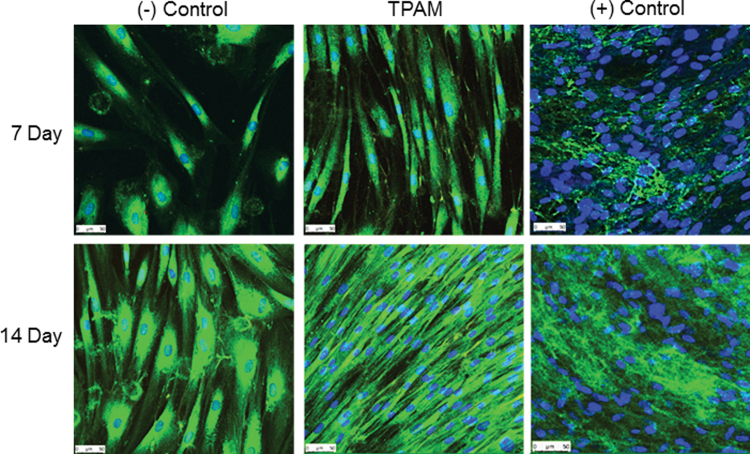
Treatment of hDF with TPAM influences collagen type 1 deposition as detected by Hoescht for nuclei (*blue*) and collagen I (*green*) stains at 7 and 14 days. Color images are available online.

The process of cell migration and proliferation within a wound bed is an important aspect of the inflammatory and proliferation phases of wound healing. TPAM extracts significantly (*p* < 0.05) promoted hDF migration in wound healing scratch assay at 20 and 27 h timepoint compared to a negative control as shown in Fig. 4. Furthermore, TPAM extracts significantly enhanced hDF proliferation (*p* < 0.0001) compared to a negative control (Fig. 5) at a 7-day time point. Once cells have migrated into the wound bed and undergo proliferation, the next phase of wound healing involves the secretion and deposition of ECM proteins. As shown in Fig. 6, TPAM extracts influenced hDF to secrete collagen type I (green).

### TPAM has immunomodulatory properties

**Figure 7.**
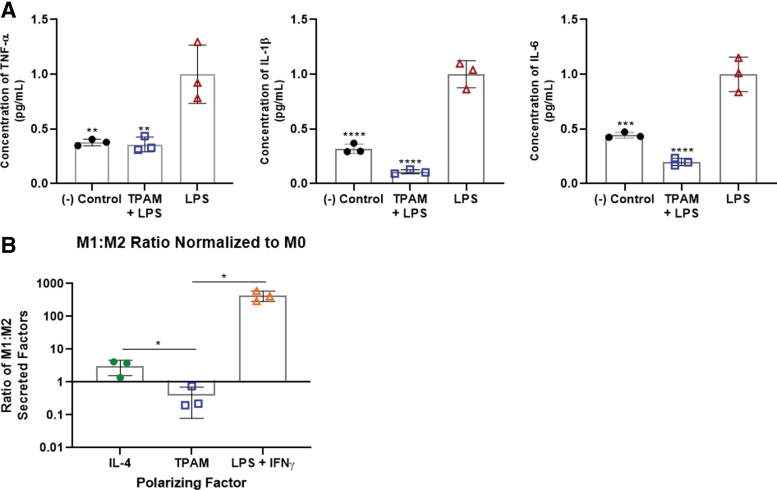
Immunological response assessment **(A)** Reduction in pro-inflammatory cytokines secreted from PBMCs in the presence of TPAM as compared to an LPS-treated positive control (One-way ANOVA, **p < 0.01, ***p < 0.001, ****p < 0.0001). **(B)** Graphical representation of the ratio of secreted M1-associated factors (IL-6, RANTES, IFNγ, TNFα) to M2-associated factors (PDGF-β, CCL-22, CCL-18, IL-18), each as normalized to the respective quantity secreted by control M0 macrophages. Secretions were collected over a 24-h window immediately following a 48-h polarization period. Control M1 and M2 macrophages were polarized with LPS/IFNγ or IL-4, respectively (*t*-test, *p < 0.05). CCL, C–C motif chemokine; IFN, interferon; IL, interleukin; LPS, lipopolysaccharide; PBMC, peripheral blood mononuclear cell; RANTES, regulated upon Activation, Normal T Cell Expressed and Presumably Secreted; TNF, tumor necrosis factor. Color images are available online.

**Figure 8.**
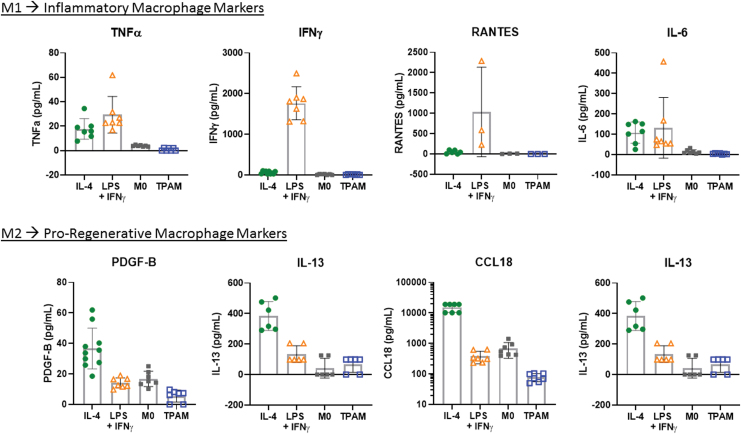
Graphs depict the absolute quantities of M1-associated (TNFα, IFNγ, RANTES, IL-6) and M2-associated (CCL22, PDGF-B, IL-13, CCL18) molecules secreted by macrophages over 24 h following polarization via M2: IL-4, M1: LPS + IFNγ, M0: basal media, TPAM: basal media +6 mm-diameter TPAM disc. Color images are available online.

Chronic wounds are often stalled in the inflammation stage of wound healing with elevated levels of pro-inflammatory cytokines. Reducing the levels of these pro-inflammatory cytokines may aid in the resolution of stalled chronic wounds. An inflammation inhibition assay was performed to evaluate the effect of TPAM extract on the levels of pro-inflammatory markers released by LPS stimulated PBMCs. As shown in Fig. 7A, TPAM extract significantly reduced the levels of pro-inflammatory cytokines, including that of TNFα, IL-1β, and IL-6 (*p* < 0.01). Additionally, the macrophage polarization assay was conducted to evaluate TPAM's influence on macrophage differentiation toward either pro-inflammatory (M1) phenotype or pro-regenerative (M2) phenotype by assessing the cytokines release in culture. As demonstrated in Figs. 7B and 8, TPAM influenced macrophages to secrete significantly higher levels of pro-regenerative cytokines (PDGF-B, CCL-22, CCL-18, IL-13) in comparison to pro-inflammatory cytokines (TNFα, IFNγ, IL-6, RANTES) yielding a significant decrease in the M1/M2 ratio to 0.39 compared to M2/IL-4 (*p* < 0.05) and M1/LPS + IFNγ (*p* < 0.01) control groups possessing average M1/M2 ratios of 3.1 and 435, respectively. These results suggest that TPAM's soluble factors may have preferentially shifted macrophage polarization toward a predominantly M2 phenotype demonstrating both its anti-inflammatory and pro-regenerative properties.

## Discussion

While placental tissues have had a role in wound healing for over 100 years, their use has expanded recently due to improved preservation methods and an increased body of evidence demonstrating clinical efficacy for wound healing, tissue repair, and regenerative properties. Preservation and manufacturing processes vary widely and can impact both ECM and biomolecule efficacy. Many single- and bi-layer membranes lack mechanical strength and degrade rapidly within wounds, requiring multiple applications. TPAM is constructed by sandwiching chorion between two layers of amnion using a dehydration process that preserves growth factors, cytokines, and ECM proteins. This unique layering results in a non-side specific application with improved handling properties. Furthermore, as demonstrated through histology and an ELISA assay, ECM and inherent bioactive molecules were well preserved in TPAM. Preservation technique along with a unique membrane construction reinforce the therapeutic potential of TPAM as a promising option for chronic wound healing.

During gestation, the placenta bears the load of pressure from the amniotic fluid and has the mechanical strength to withstand the stress induced by a growing fetus, suggesting a resilient biomaterial. The ECM components within amnion and chorion, such as collagen types I and III, are well known for their mechanical strength in connective tissues. However, processing can impact both the mechanical properties and biomolecule viability, thus highlighting the importance of attempting to preserve these properties through mild processing techniques. Our findings demonstrate that amnion and chorion contribute to the structural integrity of the membrane as determined by mechanical testing and enzyme degradation assays. TPAM has a greater maximum tensile force to break (∼6-fold change from amnion alone) and longer degradation time compared to one (∼1.8-fold change) and two (∼1.2-fold change) layered membranes. Furthermore, chorion may have a significant role in enhancing the mechanical properties and degradation times due to it being 4–5 times thicker than amnion. These findings suggest that by increasing the number of membrane layers, there is a direct correlation to increased mechanical properties that results in improved handling properties and residence time when implanted into wounds.

Growth factors and cytokines play a key role in regulating the four phases of wound healing: hemostasis, inflammation, proliferation, and remodeling. However, in chronic wounds, there is an imbalance of factors, such as an excessive pro-inflammatory cytokine presence, which contributes to an altered cellular response and ultimately stalling in the inflammation phase of healing. It has been noted that placental tissues provide factors that can help to restore the normal balance of biomolecules within the wound environment. Some of these molecules include EGF and FGF-2 that have roles in ECM deposition, neovascularization, and re-epithelialization.^28^ PDGF-AA and PDGF-AB influence the formation of granulation tissue by acting as chemoattractants and mitogens for cell division, protein synthesis, and angiogenesis.^29,30^ The three isoforms of TGF-β have overlapping functions related to angiogenesis, inflammation, ECM synthesis and deposition, and remodeling.^31^ Cytokines, also preserved in placental tissues, have roles in modulating inflammatory responses, angiogenesis, and chemotaxis.^32,33^ ECM proteins provide a favorable environment for adhesion, proliferation, and migration of cells that will initiate epithelialization, neovascularization, and resolve the chronic wound.

The data herein demonstrate the ability of soluble factors within TPAM to favorably promote fibroblast migration, proliferation, and secretion of collagen type I ECM. Fibroblasts are normally quiescent cells under homeostatic conditions, but in the presence of TPAM extracts, fibroblasts had an increased proliferation, migration, and production of collagen type I when compared to controls. Such functionalities are the hallmark of activated fibroblasts, which are also known for secreting a wide range of ECM constituents beyond collagen, promoting blood vessel formation through secretion of VEGF, and reducing total wound area through contraction of surrounding tissues, all of which are imperative for normal wound healing and a return to homeostasis.

Immune modulation is essential in the progression of normal healing. Achieving an appropriate balance of M1 and M2 macrophages is imperative for eventual cessation of the inflammation phase. This point is highlighted by Goren *et al.*'s ability to improve the re-epithelialization and overall wound closure of wounds in diabetic mice through antibody inhibition of M1 macrophages.^34–36^ Furthermore, biomaterials specifically designed to promote M2 macrophage phenotypes, whether through pore size or surface chemistry modifications, have been associated with improved vascular density, lower levels of inflammation, and enhanced rates of healing.^37,38^ While others have previously demonstrated the ability of living amniotic cells to elicit favorable M1:M2 ratios, this functionality has yet to be shown for devitalized membranes.

In this study the ability of TPAM to promote favorable M1:M2 ratios was demonstrated. M2 controls were obtained through IL-4 stimulation, thus specifically representing M2a polarization as opposed to other defined M2 subtype classifications. The ability of TPAM to lower the M1:M2 ratio compared to M2 controls is likely due to TPAM's complex array of bioactive factors known for promoting macrophage polarization along multiple different M2 lineages and thus producing a more stark display of M1 versus M2-associated secretory markers. While we only measured four distinct markers of the M2 secretome, the existence of multiple M2 subtypes implies the presence of a much wider variety of secreted factors. These include VEGF and PDGF that could promote blood vessel formation, TGF-β and insulin growth factor-1 that are known to activate fibroblasts and other somatic cells, IL-10 and IL-13 that have anti-inflammatory effects, and a subset of matrix metalloproteinases important for matrix remodeling in latter stages of healing.^35,39–41^ Furthermore, from a gross inflammatory standpoint, TPAM demonstrated an ability to effectively reduce a range of pro-inflammatory cytokines from LPS-stimulated PBMCs. These included TNFα, IL-1β, and IL-6, which are known to be secreted by immune cells including granulocytes, lymphocytes, and monocytes/macrophages and thus their reduction demonstrates TPAM's ability to modulate the behavior of multiple immune cell lineage. All of the aforementioned results suggest TPAM is a multipotent immunomodulatory construct that not only suppresses immune-mediated inflammation, but favorably modulates the phenotypes and secretions of immune cells such that pro-regenerative processes are stimulated.

Future studies will be useful to further elucidate the mechanisms underlying TPAM's demonstrated regenerative potential. Investigations into a broader variety of immune players such as T regulatory cells, Type 2 Helper T cells, and dendritic cells could provide additional valuable insights into TPAM's immunomodulatory functionalities. Further, analyzing these and other factors in more complex systems such as coculture *in vitro* fibrosis models and regenerative animal models could confer greater physiological relevance. *In vivo* models will also be valuable to evaluate the degradation profiles of various layer membranes and further validate the *in vitro* characterization work.

In summary, TPAM serves many roles in the wound healing cascade. First and foremost, our findings demonstrate that TPAM has a unique advantage over single or bi-layer membrane-based products in that it is composed of three placental membranes and thus supports improved mechanical and handling properties. On a molecular level, TPAM plays a significant role in cell behavior as demonstrated by the migration, proliferation, and ECM secretion of fibroblasts in response to the many growth factors, cytokines, and other proteins that have been preserved, are present, and active within the membrane. Furthermore, TPAM has a role in modulating the inflammatory response in cells by both reducing the amount of pro-inflammatory cytokine secretion and promotion of pro-regenerative macrophage phenotypes. These results, in combination with a previous clinical trial, suggest the unique construction of TPAM provides biophysical and biochemical attributes beneficial for supporting difficult to heal wounds through the wound healing process.^7,8^

## Innovation

TPAM is constructed utilizing a dehydration method and unique tri-layer construction, which imparts enhanced handling properties and longer degradation times, contributing to longer residence times than single- and bi-layer amnion and chorion membranes allowing for an extended release of beneficial factors for wound healing. Delivery of exogenous growth factors is shown to improve cellular migration, proliferation, ECM secretion, and immunomodulation properties. By modulating the immune response, TPAM membranes can shift a stalled chronic wound toward a pro-regenerative state. Ultimately, the use of TPAM in the treatment of complex, chronic wounds may advance the current standard of wound care treatment.

**Key Findings**

- TPAM, consisting of three layers, is a non-side specific membrane with enhanced handling properties over single- and bi-layer membranes, resulting in a slower degradation time and potentially a stronger wound barrier product.
- Inherent growth factors, cytokines, and ECM proteins such as collagen types I and III, fibronectin, and laminin are preserved in TPAM.
- TPAM extracts favorably influence fibroblast migration, proliferation, and ECM secretion and modulate inflammatory processes.

**Take-Home Messages**

- Chronic, stalled wounds have a difficult time proceeding past the inflammatory phase of wound healing if left untreated.
- TPAM provides ECM proteins, growth factors, and cytokines that have been shown to have a role in progressing difficult to heal wounds past the inflammatory phase of wound healing.
- A tri-layer construction of TPAM results in a stronger membrane, slower degradation, and enhanced handling properties, setting these apart from other placental sheet products.

## About the Authors

The authors of this article are Biomedical Engineers and are affiliated with the R&D Department of Integra LifeSciences. **Paul P. Bonvallet, PhD**, received his PhD from the department of Cell, Developmental, and Integrative Biology from the University of Alabama at Birmingham. **Sita M. Damaraju, PhD**, received her PhD in Biomedical Engineering from the New Jersey Institute of Technology and Rutgers University. **Heli N. Modi, MS**, received her MS degree in Biomedical Engineering from the New Jersey Institute of Technology. **Victoria L. Stefanelli, PhD**, received her PhD in Biomedical Engineering from the Georgia Institute of Technology. **Qiaoling Lin, BS**, received her BS in Biomedical Engineering from the Pennsylvania State University. **Sunil Saini, PhD,** currently serves as Senior Director in R&D at Integra LifeSciences, responsible for early stage product development. Dr. S.S. received his PhD in Chemical Engineering from the Georgia Institute of Technology. **Ankur Gandhi, PhD**, currently serves as a Director in R&D at Integra LifeSciences, responsible for upstream and downstream research and development. Dr. A.G. received his PhD in Biomedical Engineering from the New Jersey Institute of Technology.

## Abbreviations and Acronyms

ANOVA: analysis of variance
CCL: C–C motif chemokine
ECM: extracellular matrix
EGF: epithelial growth factor
ELISA: enzyme linked immunosorbent assay
FBS: fetal bovine serum
FDA: Food and Drug Administration
FGF: fibroblast growth factor
H&E: hematoxylin and eosin
hDF: human dermal fibroblasts
IFN: interferon
IL: interleukin
LPS: lipopolysaccharide
PBMC: peripheral blood mononuclear cell
PBS: phosphate buffered saline
PDGF: platelet derived growth factor
RANTES: regulated upon Activation, Normal T Cell Expressed and Presumably Secreted
TGF: transforming growth factor
TIMP: tissue inhibitor of metalloproteinase
TNF: tumor necrosis factor
TPAM: tri-layer placental allograft membrane
VEGF: vascular endothelial growth factor
